# Story of the Insane from Year to Year

**Published:** 1907-11-09

**Authors:** 


### THE STORY OF THE INSANE FROM YEAR TO YEAR.

### Eighteenth Annual Series.

### GLASGOW DISTRICT ASYLUM AT GARTLOCH.

This asylum contained 683 patients at the beginning of 1906 and the year closed with only one in excess of that number. There were 288 admissions, 114 recoveries, 88 discharged relieved, and 85 deaths. The total number under treatment was 971, and the average number resident was 702. The percentages of recoveries and of deaths were both rather high; the former being 39.5 per cent. of the admissions and the latter 12.1 per cent. of the average number resident. No fewer than 51 of the deaths were caused by cerebral and spinal diseases; only one by pneumonia, and eight by phthisis. This phthisical death-rate is 11.7 per cent. of the total number of deaths; and Dr. Parker remarks that since the opening of the sanatorium the number of deaths from phthisis has decreased to such an extent that the large wards in the sanatorium are no longer required for consumptives, and are now being used for senile patients and general paralytics. This shows the advantage of sanatorium treatment. "Boarding-out" has been rather largely carried out at this asylum. At present 50 cases are so treated; and this admirable system is the reason why the asylum does not show an increase of 51 patients instead of one only. Dr. Parker would go much further. He thinks that even noisy and troublesome cases might be arranged in village groups, and that under such conditions these patients would be more easily managed. He admits, however, that a medical officer and an experienced inspector would then be required; and we should fear that expense would be a bar to the common adoption of the plan. Dr. Parker would answer, that enlargements of existing asylums would not be needed; and we have so strong a dislike to those mammoth institutions that we should welcome almost any innovation that would curtail them. The annual cost of maintenance is lower by 30s. than it was for the previous year. The Visiting Committee publish no report with the general tables.

